# Allele frequencies of BRAF*V600* mutations in primary melanomas and matched metastases and their relevance for BRAF inhibitor therapy in metastatic melanoma

**DOI:** 10.18632/oncotarget.5634

**Published:** 2015-10-16

**Authors:** Imke Satzger, Lena Marks, Martin Kerick, Sven Klages, Carola Berking, Rudolf Herbst, Bernward Völker, Vivien Schacht, Bernd Timmermann, Ralf Gutzmer

**Affiliations:** ^1^ Department of Dermatology and Allergy, Skin Cancer Center Hannover, Hannover Medical School, Hannover, Germany; ^2^ Sequencing Core Facility, Max Planck Institute for Molecular Genetics, Berlin, Germany; ^3^ Department of Dermatology, Ludwig-Maximilian University of Munich, Munich, Germany; ^4^ Department of Dermatology and Allergology, HELIOS Skin Cancer Center, Erfurt, Germany; ^5^ Institute of Pathology, Nordstadt Krankenhaus, Hannover, Germany

**Keywords:** metastatic melanoma, primary melanoma, BRAF*V600* mutation, BRAF inhibitor

## Abstract

**Background:**

The detection of BRAF*V600* mutations in patients with metastatic melanoma is important because of the availability of BRAF inhibitor therapy. However, the clinical relevance of the frequency of BRAF*V600* mutant alleles is unclear.

**Patients and Methods:**

Allele frequencies of BRAF*V600* mutations were analyzed by ultra-deep next-generation sequencing in formalin-fixed, paraffin-embedded melanoma tissue (75 primary melanomas and 88 matched metastases). In a second study, pretreatment specimens from 76 patients who received BRAF inhibitors were retrospectively analyzed, and BRAF*V600* allele frequencies were correlated with therapeutic results.

**Results:**

Thirty-five patients had concordantly BRAF-positive and 36 (48%) patients had concordantly BRAF-negative primary melanomas and matched metastases, and four patients had discordant samples with low allele frequencies (3.4–5.2%). Twenty-six of 35 patients with concordant samples had BRAF*V600E* mutations, three of whom had additional mutations (*V600K* in two patients and *V600R* in one) and nine patients had exclusively non-*V600E* mutations (*V600K* in eight patients and *V600E -c.1799_1800TG > AA-* in one patient). The frequency of mutated BRAF*V600* alleles was similar in the primary melanoma and matched metastasis in 27/35 patients, but differed by >3-fold in 8/35 of samples. BRAF*V600E* allele frequencies in pretreatment tumor specimens were not significantly correlated with treatment outcomes in 76 patients with metastatic melanoma who were treated with BRAF inhibitors.

**Conclusions:**

BRAF*V600* mutation status and allele frequency is consistent in the majority of primary melanomas and matched metastases. A small subgroup of patients has double mutations. BRAF*V600* allele frequencies are not correlated with the response to BRAF inhibitors.

## INTRODUCTION

In patients with BRAF*V600E*-positive melanoma enrolled in large randomized phase III studies, treatment with BRAF kinase inhibitors such as vemurafenib and dabrafenib resulted in response rates of over 50–60% and progression-free survival (PFS) of 6–7 months. Among nonresponders in these trials, the majority had an initial period of disease stabilization, and only a minority had primary resistance to BRAF inhibitor therapy [1, 2].

Precise selection of patients is crucial for optimal use of BRAF inhibitor therapy. BRAF mutations may be detected in archived formalin-fixed, paraffin-embedded (FFPE) melanoma tissue; however, it is currently unclear whether primary tumors, consecutive metastases, or both should be preferentially analyzed because of the possibility of intertumor heterogeneity [3]. It is also unclear whether the allele frequency of BRAF*V600E* mutations is correlated with response to BRAF kinase inhibitors. Thus in the first study population, we evaluated BRAF*V600* mutations and allele frequencies in FFPE melanoma specimens using ultra-deep next-generation sequencing (NGS) and compared the results in primary melanomas and matched metastases. In a second study population we used NGS to evaluate BRAF*V600* mutations in pretreatment melanoma specimens from 76 patients with metastatic melanoma who subsequently received BRAF inhibitors, and examined correlations between BRAF*V600* allele frequencies, PFS, overall survival (OS), and objective response.

## RESULTS

BRAF*V600* mutational status was determined by ultra-deep NGS in 163 FFPE tissue samples obtained from 75 patients (Table 1 and 2). The primary melanoma and consecutive metastases from one, two, and three locations were available for 63, 11, and one patient, respectively. In addition to the 75 primary melanoma samples, the analysis included 49 skin metastases, 36 lymph node metastases, two visceral metastases, and one brain metastasis.

**Table 1:** Frequencies (%) of BRAF*V600*-mutated alleles detected by next-generation sequencing (NGS)

Patient number	Melanoma sample	NGS result	*V600E* (%)	*V600K* (%)	*V600R* (%)	*V600E2* (%)
1	pm	*V600E*	18,3	0	0	0
	ln	*V600E*	3,7	0	0	0
2	pm	*V600E*	24,4	0	0	0
	ln	*V600E*	18,7	0	0	0
	sc	*V600E*	24,6	0	0	0
	visceral	*V600E*	25,6	0	0	0
3	pm	*V600E*	45,1	0	0	0
	sc	*V600E*	17,7	0	0	0
4	pm	*V600E*	78,5	0	0	0
	sc	*V600E*	86,5	0	0	0
5	pm	*V600E*	38,5	0	0	0
	Ln	*V600E*	45,3	0	0	0
6	pm	*V600E*	40,8	0	0	0
	sc	*V600E*	33,2	0	0	0
7	pm	*V600E*	61,3	0	0	0
	sc	*V600E*	50,9	0,1	0	0
8	pm	*V600E*	9,4	0	0	0
	sc	*V600E*	7,4	0	0	0
9	pm	*V600E*	24,5	0	0	0
	sc	*V600E*	69	0	0	0
10	pm	*V600E*	10,6	0	0	1,8
	ln	*V600E*	8,1	0	0	0
11	pm	*V600E*	31,9	0	0	0
	sc	*V600E*	20,1	0	0	0
	ln	*V600E*	17,3	0	0	0
12	pm	*V600E*	23,9	0	0	0
	sc	*V600E*	47,2	0	0	0
13	pm	*V600E*	5	0,1	0	0
	ln	*V600E*	16	0	0	0
14	pm	*V600E*	29	0	0	0
	sc	*V600E*	36,2	0	0	0
	ln	*V600E*	40,7	0,1	0	0
15	pm	*V600E*	21	0	0	0
	ln	*V600E*	10,9	0	0	0
16	pm	*V600E*	51,2	0	0	0
	sc	*V600E*	24,2	0	0	0
17	pm	*V600E*	26,9	0	0	0
	sc	*V600E*	46,6	0	0	0
	ln	*V600E*	58	0	0	0
18	pm	*V600E*	23,4	0	0	0
	sc	*V600E*	54,3	0	0	0
19	pm	*V600E*	44,2	0	0	0
	ln	*V600E*	14,6	0	0	0
	sc	*V600E*	28,3	0	0	0
20	pm	*V600E*	22,4	0	0	0
	sc	*V600E*	7,1	0	0	0
21	pm	*V600E*	7	0	0	0
	sc	*V600E*	49,5	0,2	0	0
22	pm	*V600E*	31,9	0	0	0
	ln	*V600E*	34,7	0,2	0	0
23	pm	*V600E*	28,1	0,1	0	0
	sc	*V600E*	24,4	0	0	0
24	pm	*V600E*	12,7	0	0	0
	ln	*V600E*	92,2	0	0	0
25	pm	*V600E*	23,6	0	0	0
	brain	*V600E*	54,2	0	0	0
26	pm	*V600E*	5,6	0	1,4	0
	ln	*V600E+R*	20,8	0	11,4	0
27	pm	*V600K*	0,1	34	0	0
	sc	*V600K*	0	23,4	0	0
28	pm	*V600K*	0	66,2	0,2	0
	ln	*V600K*	0	9,6	0,1	0
29	pm	*V600K*	0,1	44,5	0	0
	ln	*V600K*	0	12,7	0	0
30	pm	*V600K*	0	46,5	0	0
	ln	*V600K*	0	47,8	0	0
	sc	*V600K*	0,2	50,7	0	0
31	pm	*V600K*	0,5	34,4	0	0
	ln	*V600K*	0	83,7	0	0
32	pm	*V600K*	0	32,8	0	0
	sc	*V600K+E*	13,1	19,1	0	0
33	pm	*V600K*	0	13,2	0	0
	sc	*V600K+E*	5,2	14,6	0	0
34	pm	*V600E2*	0	0	0	30,2
	sc	*V600E2*	0,1	0	0	72,9
35	pm	*V600K*	2	17,7	0	0
	sc	*V600K*	0	45,5	0	0
	sc	*V600K*	0,1	43,4	0	0
36	pm	*V600E*	5,2	0	0	0
	sc	*wild type*	0,1	0	0	0
37	pm	*V600E*	4,5	0	0	0
	sc	*wild type*	0	0	0	0
38	pm	*wild type*	0	0	0	0
	sc	*V600E*	3,4	0	0,2	0
39	pm	*V600E*	5	0	0	0
	sc	*wild type*	0	0	0	0
	sc	*wild type*	0,8	0	0	0

Three patients (26, 32, and 33) had double (BRAFV600E and non-BRAFV600E) mutations. NGS allele frequencies >3% were considered to be positive (pm = primary melanoma, ln = lymph node metastasis, sc = subcutaneous.

**Table 2:** Clinical characterization of 75 patients; 75 primary melanomas of these patients and 88 matched metastases were analyzed by ultra-deep next generation sequencing to compare BRAF*V600* status and BRAF*V600* allele frequencies of primary melanomas and matched metastases

**Clinical parameters**		
Total patients	*N*	75 (100%)
Gender	Male	46 (61%)
	Female	29 (39%)
Age (years)	Mean	61
	Median	65
	Minimum–maximum	29–91
**BRAF status**		
Tissue tested	Primary melanoma	75
	Lymph node metastases	36
	Cutaneous metastases	49
	Visceral metastases	2
	Brain metastases	1

Among the 163 tissue samples evaluated, 81 (50%) specimens were BRAF*V600*-negative, 79 (48%) specimens had a single BRAF*V600* mutation. 61 patients had BRAF*V600E (c.1799T > A)*, 16 had BRAF*V600K (c.1798_1799GT > AA)* and two patients had BRAF*V600E (c.1799_1800TG > AA*), and three (2%) specimens had two different BRAF*V600* mutations; two had BRAF*V600E (c.1799T > A)* and *BRAFV600K* (c.1798_1799GT > AA) and one had BRAF*V600E(c.1799T > A)* and BRAF*V600R (c.1798_1799GT > AT,* Table 1).

### Evaluation of BRAF status in primary melanomas and matched metastases by NGS

Consistent mutation patterns in primary tumors and matched metastatic lesions were observed in 71 of 75 (95%) patients. A total of 35 patients had concordantly BRAF-positive and 36 (48%) patients had concordantly BRAF-negative primary melanomas and matched metastases The four (5%) remaining patients each had one BRAF*V600*-positive and one BRAF*V600*-negative sample. In three of these four patients, the primary melanoma was BRAF*V600-*positive, and in the one remaining patient the metastatic tissue alone was BRAF*V600*-positive (Table 1). Of note, the BRAF*V600* allele frequencies were low (3.4–5.2%) in the positive samples from these four individuals (Table 1).

### BRAFV600E (c.1799T > A) mutations and rare mutations by NGS

Among the 35 patients with concordantly BRAF-positive samples, 26 patients had a BRAF*V600E (c.1799T > A)* mutation in both the primary melanoma and consecutive metastases, eight patients had BRAF *V600K* (c.1798_1799GT > AA) mutations (eight primary melanomas, four lymph node metastases, and four skin metastases), and one patient had a BRAF*V600E (c.1799_1800TG > AA*) mutation (in the primary melanoma and matched skin metastasis) (Table 1). Three metastatic specimens from these 35 patients (two skin metastases and one lymph node metastasis) showed BRAF*V600K* (c.1798_1799GT > AA, *n* = 2) or BRAF*V600R (c.1798_1799GT > AT;* n = 1) mutations with an allele frequency >3%, in addition to BRAF*V600E (c.1799T > A)*.

### Frequencies of mutated BRAFV600 alleles in primary melanomas and matched metastases by NGS

**Figure 1:**
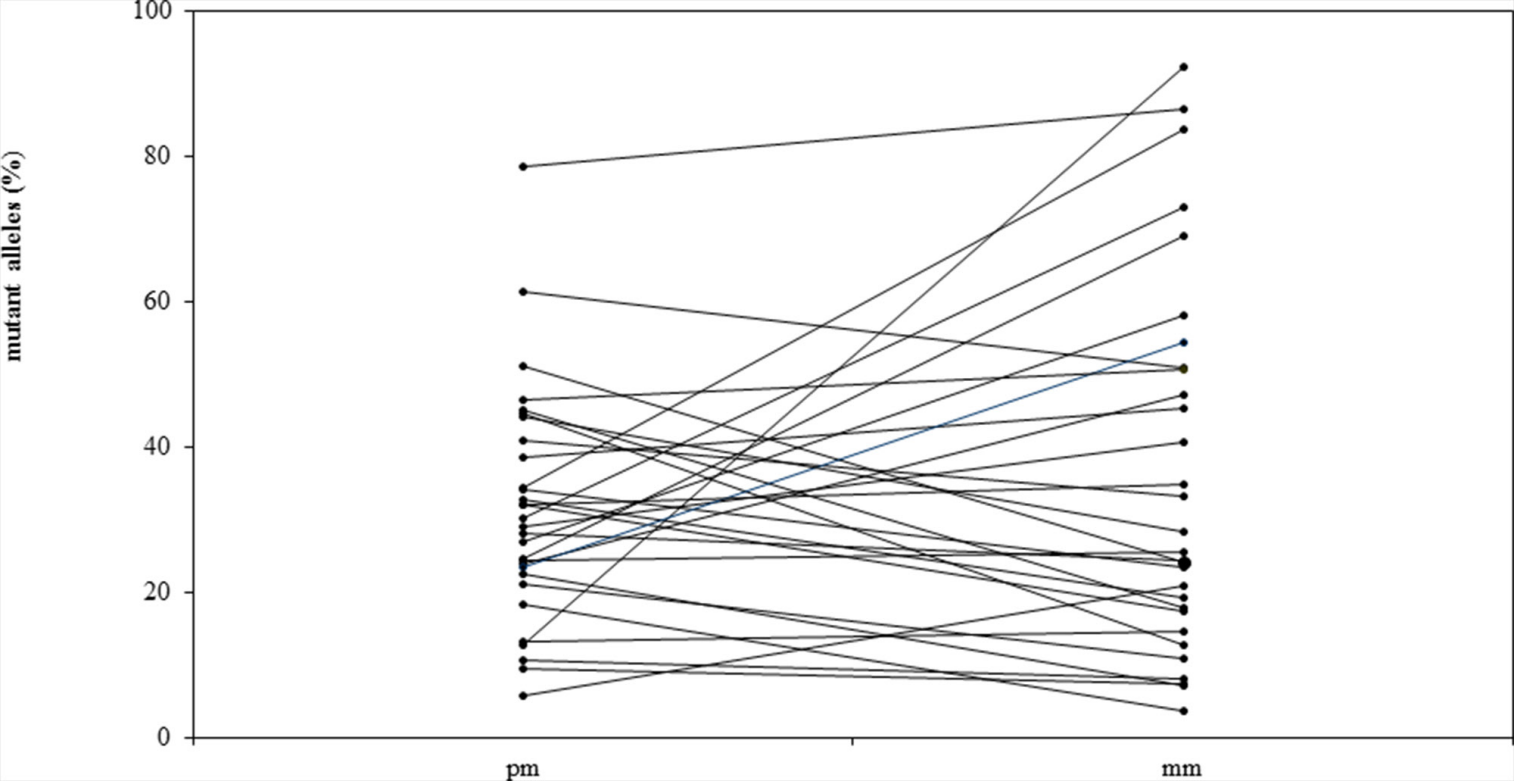
Allele frequencies (%) of BRAF*V600* mutations in primary melanomas (pm) and matched metastases (mm) in 35 patients with metastatic melanoma

The median percentage of mutated alleles was 28% in primary melanomas and 26% in consecutive metastases (Figure 1). The mutant allele frequency was higher in the primary melanoma than in the metastases in 16 patients, and higher in the metastases than the primary melanoma in 19 patients (Table 1, Figure 1). In 27 of 35 (77%) patients with BRAF*V600* mutations, the percentage of mutated alleles in the primary melanoma and metastases differed by <3-fold. In the eight patients in whom the percentage of mutated alleles in the primary melanoma and metastases differed by >3-fold, the frequencies of mutated alleles was higher in the primary melanoma in four patients and higher in the metastases in four patients. The differences in allele frequencies between primary and metastatic tissue in six of these eight patients could be attributed to differences in tumor cell content in the various tissues.

### Allele frequencies of patients treated with BRAF inhibitors and their impact on therapy outcome

Pretreatment samples from 76 patients with BRAF*V600E*-positive metastatic melanoma who were treated with the BRAF inhibitors vemurafenib (*n* = 67) or dabrafenib (*n* = 9) were retrospectively analyzed by NGS. The baseline characteristics and response to therapy after a mean follow-up of 11.4 months are summarized in Table 3. The available samples included nine primary melanomas, 29 lymph node metastases, 28 cutaneous or subcutaneous metastases, eight visceral metastases, and two brain metastases. As shown in Table 3, BRAF*V600E (c.1799T > A)* allele frequencies in pretreatment melanoma tissue were ≤5% in two patients, >5–10% in four patients, >10–15% in three patients, >15–20% in 11 patients, > 20–25% in 6 patients, >25–50% in 33 patients, and >50% in 17 patients.

**Table 3:** Characterization of 76 patients with BRAF*V600E* mutations who were treated with BRAF inhibitors for metastatic melanoma

**Clinical parameters**		
Total patients	*N*	76 (100%)
Gender	Male	41 (54%)
	Female	35 (46%)
Age (years)	Mean	56
	Median	60
	Minimum–maximum	13–84
**BRAF status**		
Tissue tested	Primary melanoma	9 (12%)
	Lymph node metastases	29 (38%)
	Cutaneous metastases	28 (37%)
	Visceral metastases	8 (10%)
	Brain metastases	2 (3%)
Allele frequencies	Mean	31.9
BRAF*V600E* mutation (%)	Median	34.1
	Minimum-Maximum	3.7–81.2
	≤5	2 (3%)
	>5–10	4 (5%)
	>10–15	3 (4%)
	>15–20	11 (15%)
	>20–25	6 (8%)
	>25–50	33 (43%)
	>50	17 (22%)
**Treatment**		
BRAF inhibitor	Vemurafenib	67 (88%)
	Dabrafenib	9 (12%)
Prior therapies for metastatic melanoma	No	44 (57%)
	1 prior therapy	12 (16%)
	2 prior therapies	14 (19%)
	3 prior therapies	3 (4%)
	≥4 prior therapies	3 (4%)
**Response to treatment**		
Best response	CR	5 (7%)
	PR	40 (53%)
	SD	17 (22%)
	MR	10 (13%)
	PD	4 (5%)
Time to progression (months)	Mean	5.5
	Median	7.1
	Minimum–maximum	0.5–26.1
Progression	No	15 (20%)
	Yes	61 (80%)
**Follow-up**		
Death	No	31 (41%)
	Yes, due to melanoma	41 (54%)
	Yes, other cause	4 (5%)
Follow-up (months)	Mean	11.4
	Median	9.7
	Minimum–maximum	0.8–27.8

(sc = subcutaneous, CR = complete response, MR = mixed response, PD = progressive disease, PR = partial response, SD = stable disease).

**Figure 2:**
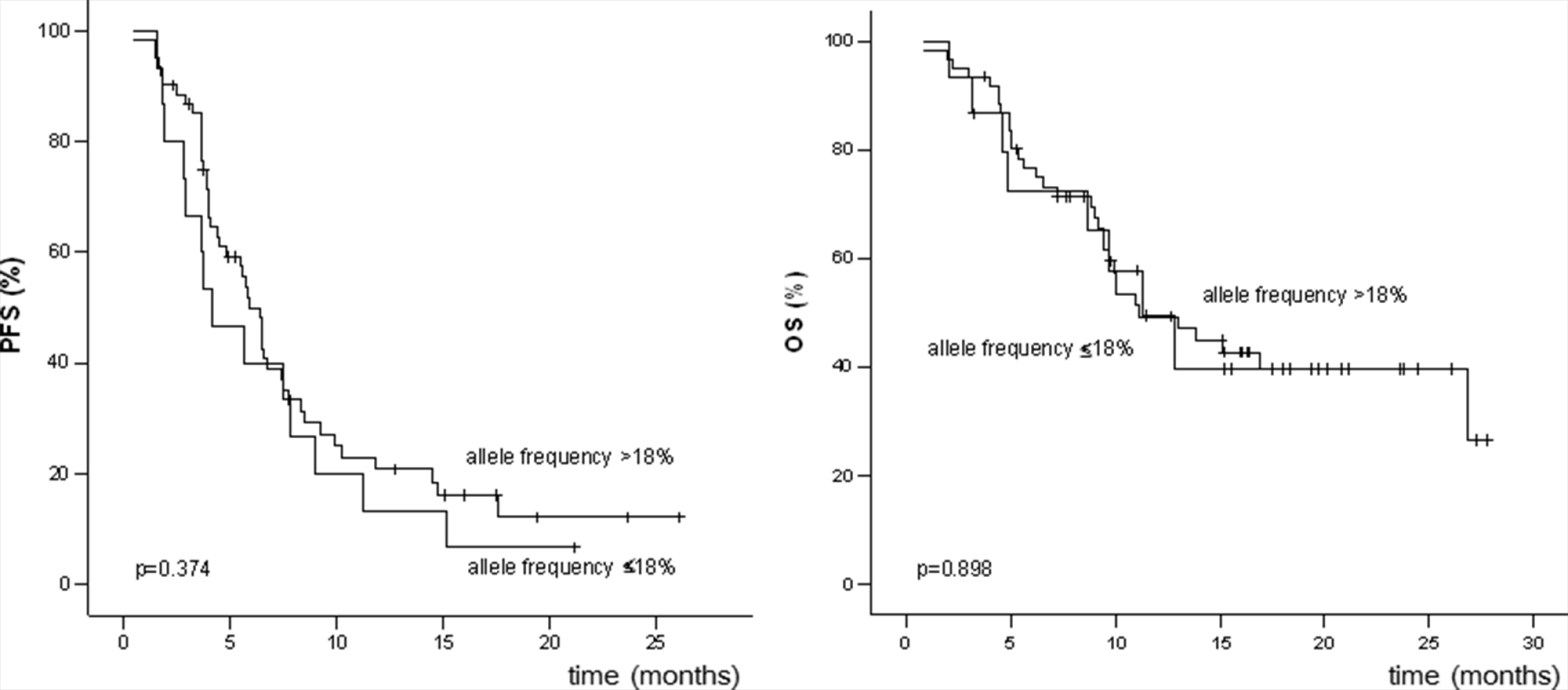
Progression-free survival (PFS) and overall survival (OS) of patients with BRAFV600 allele frequencies ≤18% and >18%

Allele frequencies were not significantly correlated with either PFS or OS in Kaplan-Meier analyses in which different cut-offs were used (≤15%, ≤18%, ≤20%, and ≤25%). Comparisons of PFS and OS in patients with allele frequencies ≤18 and >18% are shown in Figure 2 (*p* = 0.374 for PFS and *p* = 0.898 for OS). Odds Ratio was calculated to determine the magnitude of differences that can be detected with this relatively small cohort (Table 4).

**Table 4:** Required 95% confidence interval limits for statistical significance in response analysis

Parameters	Cut-offs			
	15%	18%	20%	25%
Proportion π_1_	15%	25%	28%	31%
Proportion π_2_	61%	64%	64%	64%
Odds ratio^1^	8.86	5.33	4.57	3.96
**Distance**–^2^	**1.89**	**1.28**	**1.12**	**1.01**
**Lower limit**^3^	**1.33**	**1.48**	**1.49**	**1.44**
**Upper limit**^3^	**58.93**	**19.19**	**13.99**	**10.89**

Note: π1: proportion of responders with BRAFV600E allele frequency ≤ cut-off; π2: proportion of responders with BRAFV600E allele frequency > cut-off.

1 This is the minimum required effect size estimated for power equal to 80% (proportion of responders with BRAFV600E allele frequency > cut-off against proportion of responders with BRAFV600E allele frequency ≤ cut-off).

2 Distance refers to the difference between the logarithm of odds ratio and its lower limit.

3 Results are presented in the exponential (original) scale.

The response to therapy (complete response [CR] and partial response [PR] versus stable disease [SD], mixed response [MR], and progressive disease [PD]) was not significantly correlated with allele frequencies in univariate analyses in which different cutoffs were used (15%, 18%, 20%, and 25%). A total of 9, 15, 20, and 26 patients had BRAF*V600E* allele frequencies ≤15%, ≤18%, ≤20%, and ≤25%, respectively. Response rates (CR and PR) in these categories were as follows: BRAF*V600E* allele frequency ≤15% versus >15% (44% and 61%, *p* = 0.473); ≤18% versus >18% (40% and 64%, *p* = 0.142); ≤20% versus >20% (45% and 64%, *p* = 0.185); and ≤25% versus >25% (50% and 64%, *p* = 0.326).

## DISCUSSION

Since the discovery of BRAF mutations in melanoma in 2002 [4], advances in molecular characterization of the disease have led to the development of specific BRAF inhibitors that are now available for the treatment of patients with metastatic melanoma. In the first part of this study, we used ultra-deep NGS to assess the BRAF*V600* mutation status of primary melanomas and matched metastases and their influence on the outcome of BRAF inhibitor therapy.

For the first time, we showed that type and frequency of BRAF*V600* mutations are consistent in the primary melanoma and matched metastases in the majority of patients. In most patients with discordant BRAF*V600* status, in which one tissue sample was positive and one sample negative, this could be explained by low allele frequencies where the allele frequency was close to the threshold of detection in the positive sample but below the threshold of detection in the negative sample. Differences in allele frequencies could also be explained by differences in tumor cell content in the specimens. These results suggest that, in the majority of melanoma cases, the BRAF mutation status of the primary tumor is retained in metastases, and that primary and/or metastatic tissue can be used for routine mutational analysis provided that sufficient tumor cell content is available.

Five recently published studies have analyzed the BRAF mutation status of melanoma samples with different molecular methods (Table 5). Consistent with our observations, these analyses also found the mutational status of primary tumor and metastatic tissue to be concordant in the majority of cases. Intrapatient homogeneity of BRAF mutations has also been reported in all patients (*n* = 64) included in a recent study that used immunohistochemical methods for detection of BRAF mutants [5].

**Table 5:** Evaluation of BRAF*V600* mutations in primary melanomas and matched metastases by different molecular detection methods

Detection method	Tumor cell content (%)	Concordance rate, n/N (%)	Ref.
NGS	>30	71/75(95)	This study
HRM + direct sequencing (Sanger)	>10	84/88(95)	(12)
MS-PCR + direct sequencing (Sanger)	<33 to >67	10/18 (56)	(3)
Pyrosequencing	>75	43/53(81)	(14)
Direct sequencing (Sanger)	NS	21/24(87)	(13)
Direct sequencing (Sanger)	≥80	87/102(85)	(16)

(HRM = high-resolution melting curve analysis, MS-PCR = mutagenically separated polymerase chain reaction, NGS = next-generation sequencing, NS = not stated)

Our findings suggest that melanomas can be heterogeneous with regard to BRAF*V600* mutations. The allele frequency was below 50%, which would be the expected result if cells show a consistent heterozygous mutation. For example, patient number 2 showed allele frequencies that ranged from 18.7% to 25.6% in the primary tumor and three metastatic samples (Table 1). The results of a study that used a BRAF*V600E*-specific antibody and showed heterogeneous staining in 13 of 58 (22%) melanoma samples provides further evidence of heterogeneity of BRAF*V600* mutations [6]. Moreover, a further study that used single cell suspensions to assess BRAF mutations found 9 of 10 primary melanomas and 0 of 3 metastases to be heterogeneous [7].

Despite these data that suggest BRAF*V600* mutations are heterogeneous, the clinical response to BRAF inhibitors is homogenous [1, 2] and suggests that the BRAF*V600* mutation is relevant in the majority of melanoma cells.

Approximately 10% of patients with BRAF *V600* mutations had double mutations, comprised of the BRAF*V600E* and an additional rare mutation. This phenomenon has not been reported previously and demonstrates the ability of NGS to detect different mutations that are difficult or impossible to detect with techniques used in other studies, such as Sanger sequencing, pyrosequencing or melting curve analyses [3, 8–11]. This phenomenon may also be the result of intratumor heterogeneity.

Allele frequencies had no impact on PFS, OS, and objective response rates in the 76 patients with BRAF*V600E*-positive metastatic melanoma treated with BRAF inhibitors. Although the usual precautions regarding retrospective analyses apply, the median PFS of 7.1 months and the response rate of 60% are very close to the results of the phase III studies [1, 2], supporting the validity of our findings. These results are also in line with the recently published observation of Wilmott et al. that the intensity and distribution of BRAF*V600E* immunohistochemical staining is not correlated with clinical outcomes [6].

The observation that patients with allele frequencies <18% and ≥18% have similar PFS and OS is clinically relevant, because an allele frequency of 18% is around the detection limit of Sanger sequencing [12], which is commonly used in routine clinical practice. Thus, in the case of a negative Sanger sequencing result, our data suggest that retesting with a more sensitive assay may be worthwhile to detect alleles that may be present at lower frequencies.

In conclusion, we show here that BRAF*V600* mutation status and allele frequency are consistent in the majority of primary melanomas and matched metastases, that a subgroup of patients has double mutations, and that the allele frequency of melanoma tissue is not correlated with treatment response in our patient cohort.

## MATERIALS AND METHODS

### Patients

In the first study population we retrospectively analyzed melanoma samples from patients with metastatic melanoma who were treated at the Skin Cancer Center Hannover, Germany, between 1995 and 2011. Patients were selected for this study on the basis of availability of primary melanoma tissue and tissue from at least one matched metastasis.

In a second different study population, BRAF*V600* allele frequencies were determined by NGS in pretreatment melanoma tissue specimens from metastatic melanoma patients who were treated with BRAF inhibitors at three German skin cancer centers (Munich, Erfurt and Hannover). Patients had been treated with standard dosages of vemurafenib (960 mg b.i.d.) in a phase III clinical trial [13] or expanded access program, or dabrafenib (150 mg b.i.d.) in phase III clinical trials [14]. Only patients with BRAF*V600E* mutations and with pretreatment FFPE tumor specimens available for NGS analysis were included. Patients with non-*V600E* mutations and patients with double mutations were excluded.

Response to therapy in the clinical trials was assessed with computed tomography every 8–12 weeks, as required by the protocols [13, 14]. Tumor responses were determined according to Response Evaluation Criteria In Solid Tumors (RECIST) version 1.1 [15].

### Histopathology, macrodissection, DNA extraction

One slide at the beginning of each serial section was stained with hematoxylin-eosin and histopathologically examined to determine the tumor cell content. Only samples with a tumor cell content of at least 30% were included in this study. The area of interest was circled on the stained slide and macrodissection was performed on the corresponding unstained slides using a scalpel. DNA was extracted using the cobas^®^ DNA Sample Preparation Kit (Roche, Grenzach-Wyhlen, Germany).

### Ultra-deep NGS

Isolated DNA (350–976 ng) from all FFPE specimens was subjected to analyses by NGS using a Roche GS Junior System to detect BRAF*V600* mutations on exon 15. The NGS procedure was done according to the manufacturer's specifications [16]. Amplicon processing, library preparation and emulsion PCR were done according to the manufacturer's directions for the GS Junior Titanium Series (Roche). Around 500,000 enriched beads were loaded on a 454 Junior Sequencer (Roche, Basel, Switzerland). Demultiplexing and variant calling was done with the Amplicon Variant Analyzer v2.7 software from Roche. The average sequencing coverage of BRAF was >5000. The presence of a BRAF*V600* mutation was defined as the presence of a non-reference base in a minimum of 3% of reads.

### Approval by ethics committee

The collection of clinical and follow-up data, performance of mutational analyses, and correlation with clinical data was approved by the Ethics Committee of Hannover Medical School (vote 1849-2013).

### Statistical analyses

Associations between BRAF*V600E* allele frequencies and clinical outcomes were tested by log-rank test (Kaplan-Meier analyses) using different allele frequencies as cutoffs (15%, 18%, 20%, and 25%). Qualitative comparisons of objective response to therapy with BRAF inhibitors were performed using RECIST 1.1 criteria. In these comparisons, responders were defined as having either a CR or PR and nonresponders were defined as having SD, an MR, or PD. Analyses of responses to therapy (response versus nonresponse) and allele frequencies were performed by using a two-tailed Fisher's exact test. Statistical significance was defined as an alpha level <0.05. SPSS 19.0 (SPSS Inc; Chicago, IL, USA) was used for Kaplan-Meier tests, Fisher's exact test and calculation of Odds ratio (OR).
